# A Novel Connectivity-Based LEACH-MEEC Routing Protocol for Mobile Wireless Sensor Network

**DOI:** 10.3390/s18124278

**Published:** 2018-12-05

**Authors:** Muqeet Ahmad, Tianrui Li, Zahid Khan, Faisal Khurshid, Mushtaq Ahmad

**Affiliations:** School of Information Science and Technology, Southwest Jiaotong University, Chengdu 611756, China; zahid@my.swjtu.edu.cn (Z.K.); faisalnit@gmail.com (F.K.); mushtaq.ahmad91@gmail.com (M.A.)

**Keywords:** cluster-leader (CL), mobile wireless sensor node (MWSN), mobile energy efficient and connected (MEEC), reference point group mobility model (RPGM)

## Abstract

In mobile wireless sensor network (MWSN), the lifetime of the network largely depends on energy efficient routing protocol. In the literature, cluster leader (CL) is selected based on remaining energy of mobile sensor nodes to enhance sensor network lifetime. In this study, a novel connectivity-based Low-Energy Adaptive Clustering Hierarchy-Mobile Energy Efficient and Connected (LEACH-MEEC) routing protocol was proposed, where CL is selected based on connectivity among neighboring nodes and the remaining energy of mobile sensor nodes. Consequently, it improves data delivery, network lifetime and balances the energy consumption. We studied various performance metrics including the number of alive nodes (NAN), remaining energy (RE) and packet delivery ratio (PDR). Our proposed LEACH-MEEC outperforms all other algorithms due to the connectivity metric. Moreover, the performance of mobility models was investigated through graphical and statistically tabulated results. The results show that Reference Point Group Mobility model (RPGM) is better than other mobility models.

## 1. Introduction

The Internet of Things (IoT) is a dynamic wide-ranging network that includes varieties of mobile and static sensors, data gathering devices such as global positioning system (GPS) sensors, radio frequency identification (RFID) sensors, and laser and infrared sensing scanners that are connected through the Internet and exchange information with each other based on an agreement [1,2]. Wireless sensor network (WSN) is a group of static sensor nodes which gather information and deliver to the base station. The data gathered by some static sensor nodes can be imprecise and it may suffer network failure, which affects connectivity and reliability of the network. Hence, it has limited applications. Due to fast development in IoT and mobile Internet technology, mobile wireless sensor network (MWSN) has become a popular field of research and replacement of static WSN. MWSN is a collection of tiny mobile sensor motes that aim to sense data from the environment and effectively deliver the base station. These mobile motes maintain the communication links among the neighboring nodes while collecting and processing the data for efficient communication, enhancing the network performance [3]. MWSN can be classified into three types [4], which are as follows.

- Mobile base station and mobile sensor nodes
- Mobile base station and static sensor nodes
- Static base station and mobile sensor nodes

The main difference between MWSN and WSN is sensor node mobility, which enhances the connectivity, network adaptability, and the reliability of the sensor networks. Simultaneously, MWSN stabilizes the energy utilization and improves the lifetime of the sensor network [1]. However, due to frequent changes in topology and complex environment in different applications, MWSN faces challenges related to energy efficiency, data delivery and data aggregation [5,6]. MWSN has a wide range of applications that depend on environment and setup. Space mission sensor robots [7], border monitoring mobile sensors [8], flying bee robot-sensors (ROBOBEE) [9] and undersea submarines movement detection by mobile sensor nodes [10] are common examples of MWSNs.

Routing is the core aspect of all sorts of networks, which is used to send the (sensed) data between mobile sensor nodes and base stations. This requires an efficient and reliable communication. In the literature, several routing schemes have been studied for WSN, e.g., flooding, multipath, Quality of Service (QoS), hierarchical and geographical routing, etc. Recently, much work has been done on hierarchical based routing protocols for static and mobile networks. Since it chooses a comparatively short path for routing, it is resistant to failures and efficient in the use of remaining energy. Then, it has a low overhead and enhances network lifetime [11,12]. In a hierarchical architecture, sensor nodes are classified into optimal groups based on their homogeneous properties. Afterwards, these group members select a Cluster Leader (CL) that helps member nodes transfer sensed data to the base station.

**Figure 1:**
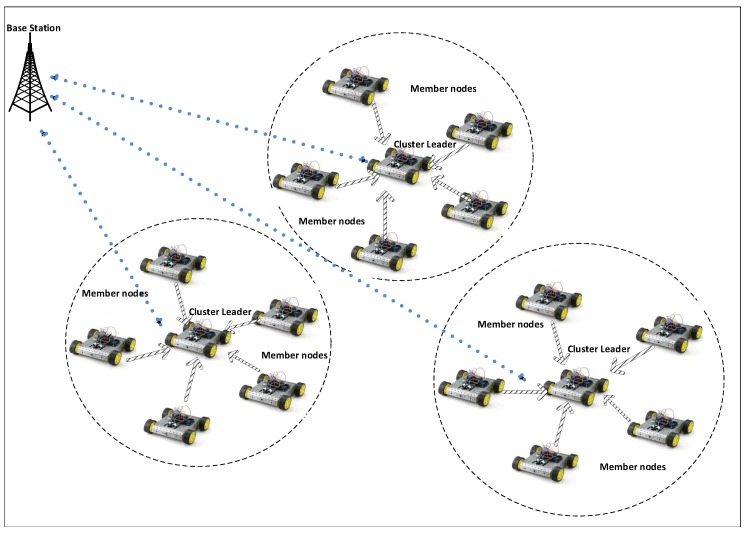
An example of Mobile LEACH routing protocol.

Low-Energy Adaptive Clustering Hierarchy (LEACH) is a basic energy efficient routing protocol of hierarchical routing protocol’s family [13], where the sensor nodes remain static. Hence, it achieves higher energy efficiency but has limited applications. However, mobile sensors have several applications but also bring many challenges such as energy depletion and squatter network lifetime. LEACH-Mobile is the first mobility-based LEACH routing protocol [14], where CL has to wait for two successive time division multiple access (TDMA) failed cycles before declaring a mobile node as a non-member. Although such a mobile node may become a member of another CL, data for two TDMA slots is lost. Consequently, this approach increases overhead and reduces energy efficiency. Figure 1 shows an example of a single hop mobile LEACH routing protocol, where a base station is static and sensor nodes are mobile.

The role of mobility models in the performance of MWSN is of enormous importance. Mobile sensor node distribution and selection of an appropriate mobility model not only improve the overall performance, but also improve the clustering process of a routing protocol [15,16]. To determine the best mobility model in a specific WSN application is still a complex issue. The mobility model includes movement order for MWSN, location, and acceleration showing maximum and minimum velocity over time. Therefore, it will help to evaluate the performance of MWSN routing protocols. In [17], effect of mobility models on Distance Vector (DV)-hop based localization algorithm is discussed. In [18], the performance of Routing Protocol for Low power and Lossy network (RPL) routing algorithm is analyzed on three different mobility models. There are various mobility models, but, in this study, we considered four mobility models: Reference Point Group Mobility model (RPGM), Random Waypoint Mobility Model (RWP), Gauss–Markov Mobility Model (G-M) and Manhattan Grid Mobility Model (MG) [19]. In the areas such as battlegrounds, disaster relief mission and other hazardous missions, where mobile sensor nodes have got their applications, each member (mobile sensor) node has to follow a group leader to achieve successful completion of the mission [1]. In RWP model [20], the nodes move in random order within the simulation area without following any group leader. In GM model [21], the values of direction and speed of any node at a particular time are updated depending upon immediate previous value. In MH model [22], the simulation area is split into horizontal and vertical path lines. Each node has to change direction (it can turn left, turn right or go straight) at intersection point depending upon probabilistic value. In RPGM mobility model, which is quite similar to clustering process, each group of nodes has a logical center (group leader) along with member nodes [17,23]. RPGM is used to simulate real time applications such as battle field, border monitoring, and disaster relief mission where mobile sensor nodes need to move in the form of group while following a movement of the group leader [24]. Consequently, RPGM shows higher connectivity than other mobility models [25,26].

Sensor node connectivity is specified as a ratio of number of sensor nodes that can actively communicate with the base station to the total number of sensor nodes. In MWSN, connectivity and coverage factors are interrelated. An ensured connectivity with a dynamic coverage is required for efficient sensing of any event. Connectivity depends upon several aspects such as sensor node distribution, communication energy, mobility, distance between sensor nodes, signal dissemination medium, signal dissemination loss, etc. [27].

Due to mobility, sensor nodes alter their positions after initial distribution. Sensor node mobility and node failure affect the transmission path, which impacts connectivity in MWSN [28]. Unplanned mobility can create coverage problem [29], whereas planned mobility (mobility models) can be applied to improving connectivity and enhancing lifetime of network [30]. To best of our knowledge [31], only two connectivity based LEACH algorithms have been proposed thus far: LEACH based on Density of node distribution (LEACH-D) [32] and Orphan- LEACH (O-LEACH) [33]. However, both studies are proposed for static sensor nodes.

In this paper, a novel connectivity based LEACH-Mobile Energy Efficient and Connected (LEACH-MEEC) algorithm is proposed. The binary disk sensing model is used to calculate neighborhood density. We propose a probabilistic connectivity model to compute connectivity among neighboring nodes. The main contributions of this paper are given as below.

- We propose LEACH-MEEC, where the connectivity and remaining energy of mobile sensor nodes are used as metrics for CL selection after the first round and onwards. This proposed metric significantly improves the performance as compared with the existing schemes.
- The proposed LEACH-MEEC is analyzed under different mobility models, using eight datasets with two different speed levels.

The rest of paper is organized as follows. Section 2 includes the related work. Section 3 discusses the proposed framework of LEACH-MEEC. Section 4 presents the simulation and results. Section 5 concludes the paper.

## 2. Related Work

A heterogeneous mobile LEACH protocol is proposed in [34]. It contains static sensor nodes with mobile base stations where CL is selected based on probability function and data are transferred to base station based on energy function. A mobility factor parameter is introduced for the CL election by initial mobile LEACH-Mobile-Enhanced (LEACH-ME) routing protocol [16]. However, this protocol increases complexity and energy depletion but performs well at a high mobility. Another mobility-based clustering (MBC) protocol was proposed by Deng et al. [35], who used two metrics for the selection of CL, i.e., remaining energy and node speed. It has applications for large-scale networks but there is a rapid change in distance between nodes due to the high mobility. CL node may select a member node that has maximum remaining energy and mobility factor, but it may have a maximum distance from the CL node as well. Consequently, it drains CL energy. A mobile LEACH algorithm for large-scale networks was proposed by Souid et al. [36], where energy is considered as the main component, defining three levels of energies with round time length. However, it has applications for only small scale static sensor network. A energy efficient LEACH-1R was proposed by Khushbu and Khunteta [37], where the CL selection is performed after the first round, only if the remaining energy is less than the threshold value. However, the author did not specify the mobility model. Similarly, LEACH-Centered Cluster-head (LEACH-CCH) was proposed by Corn and Bruce [38], where the energy utilization is reduced by predicting the positions of mobile sensor nodes and reconstructing clusters accordingly. However, the nodes distribution is performed randomly and the mobility model is not mentioned. A LEACH-Mobile Average Energy (LEACH-MAE) based routing protocol is proposed in [39], which selects CL based on remaining energy metric. Here, a CL can add member nodes in a cluster that may have maximum remaining energy, but their distance from the CL node may also be maximum. Hence, CL node may lose a lot of energy to aggregate data from member nodes. LEACHDistance-M [40] is proposed for MWSN, where the selection of CL is based on remaining energy, lower-upper threshold distance and minimum mobility. However, it only assumes 30% of sensors nodes are mobile, while remaining sensor nodes are static.

Those above-mentioned approaches [35,36,37,38,39] elect CL based on residual energy. The importance of energy metric in MWSN is vital, however, relative position, radio coverage and spatial density of mobile sensors are also important metrics for stability, consistency, and reliability of the CL, respectively. Connectivity in MWSN is a function of three important factors: transmission range, sensor speed and spatial density [41,42]. Therefore, connectivity among nodes achieves robust communication, energy efficiency, reduced communication overhead and network scalability. The concept of connectivity in WSN is used in different perspectives. In [43], Abdel-Mageid et al. improved connectivity using potential field theory and local virtual force to calculate mobility and location among mobile neighbor nodes. The conditional connectivity based algorithms were discussed in survey article [44], where the radio range were considered larger than twice of sensing range, consequently they achieved high connectivity at the cost of energy efficiency. To the best of our knowledge, connectivity as a metric for CL selection in mobile LEACH is unexplored, however, connectivity is discussed in MWSN in different perspectives. Hence, the motivation of this work is to combine connectivity with remaining energy in CL selection.

## 3. Proposed Framework

This section highlights preliminaries, node distribution, energy model and connectivity model for LEACH-MEEC routing protocol. In LEACH-MEEC, we consider:

- Location of base station is anchored and positioned outside the area of sensors distribution.
- The *N* sensor nodes are distributed randomly.
- All sensors are homogeneous in nature, having similar specification.
- Mobile sensor nodes can move randomly with a specified speed following a certain mobility model pattern.
- Mobile sensor nodes can communicate directly with base station.

### 3.1. Mobile Sensor Distribution

We consider that the *N* sensor nodes are deployed randomly in a square field (100 × 100 m${}^{2}$) following Gaussian distribution method. Equation (1) defines the probability density function (PDF) of node distribution.

(1) $$f\left(N_{xi},N_{yi}\right)=\frac{1}{2\pi \sigma_{N_{xi}}\sigma_{N_{yi}}}e^{-\left(\left(\frac{{\left(N_{xi}-N_{xj}\right)}^{2}}{2\sigma_{N_{xi}}^{2}}\right)+\left(\frac{{\left(N_{yi}-N_{yj}\right)}^{2}}{2\sigma_{N_{yi}}^{2}}\right)\right)},$$

where $N_{xj}$ and $N_{yj}$ are the positioning point, and $\sigma_{N_{xi}}$ and $\sigma_{N_{yi}}$ are the standard deviations for $N_{xi}$ and $N_{yi}$ dimension, respectively [45]. Each sensor node follows a normal distribution with different means and variances, due to different locations and random movement dependent upon mobility models, as shown in Figure 1. Let $N_{xj},N_{yj}$ deployment point be (0,0). Then, Equation (1) can be re-written as,

(2) $$f\left(N_{xi},N_{yi}\right)=\frac{1}{2\pi \sigma_{N_{xi}}\sigma_{N_{yi}}}e^{-\left(\left(\frac{{\left(N_{xi}\right)}^{2}}{2\sigma_{N_{xi}}^{2}}\right)+\left(\frac{{\left(N_{yi}\right)}^{2}}{2\sigma_{N_{yi}}^{2}}\right)\right)}$$

### 3.2. Energy Model

In this paper, three levels of energy consumption model are considered for mobile sensor node, i.e., wireless radio antenna’s transmission energy $E_{Tran}$, receiver energy $E_{Recv}$ [2,46] and mobility energy $E_{mob}$. Equation (3) calculates transmission energy $E_{Tran}$ to transmit *s* bit message over a specific distance $d_{is}$.

(3) $$E_{Tran}\left(s,d_{is}\right)=sE_{Tran\_elec}+sE_{Tran\_amp}{d_{is}}^{\varphi },$$

where $E_{Tran\_elec}$ is a radio dissipation energy and $E_{Tran\_amp}$ energy is transmission circuitry energy constant (their values are defined in simulation parametric Table 1). φ is a path loss exponent and it depends on $d_{is}$, which is the distance between the transmitting and receiving nodes. The distance $d_{is}$ is compared to distance $d_{m}$. In Equation (4), $d_{m}$ is calculated, where $h_{r}$ and $h_{t}$ are the height of antennas, SL is the loss of system, $pi_{val}$ is known as Archimedes’ constant (that is approximately equal to 3.14) and λ is wavelength of the signal.

(4) $$d_{m}=\frac{4pi_{val}\sqrt{SL}h_{r}h_{t}}{\lambda }$$

If $d_{is}<d_{m}$, then the free space model is ideal $\left(\phi =2,E_{Trans\_amp}=friss\right)$. If $d_{is}$>$d_{m}$, then the multipath model is selected $\left(\phi =4,E_{Trans\_amp}=two\_ray\_amp=multipath\right)$. The receiver energy $E_{Recv}\left(s\right)$ is defined as the energy requires to receive *s* bit message by receiving node. It requires $sE_{Recv_{e}lec}$, which is the energy spent by electric circuit of antenna to receive *s* bit message, and $E_{\left(idle+error\right)}$ idle nature of receiver and error term are assumed as constant value of energy loss (due to obstacle blocking line of sight between two sensor nodes). It is given as follows:

(5) $$E_{Recv}\left(s\right)=sE_{Recv\_elec}+E_{\left(idle+error\right)}$$

The energy dissipation due to the mobility of sensor nodes is denoted by $E_{Mob}$. A mobile sensor node movement between two points cost $E_{Comp}\left(N_{i}\right)$ computational energy and $E_{mov}\left(N_{i}\right)$ energy loss due to mobility for each round. It can be decomposed as follows.

(6) $$E_{Mob}\left(N_{i}\right)=E_{Comp}\left(N_{i}\right)+E_{mov}\left(N_{i}\right),$$

(7) $$E_{Comp}\left(N_{i}\right)=\Delta {Ic}_{T_{m}}(N_{i})*\Delta Vt_{T_{m}}\left(N_{i}\right)*T_{m},$$

where $E_{Comp}\left(N_{i}\right)$ is the dissipation of computational energy due to transmission of one bit in $T_{m}$ seconds [47]. $E_{mov}\left(N_{i}\right)$ is the depletion of energy due to mobility of nodes in $T_{m}$ seconds. $\Delta {Ic}_{T_{m}}\left(N_{i}\right)$ and $\Delta Vt_{T_{m}}\left(N_{i}\right)$ are given as follows:

(8) $$\Delta I_{T_{m}}\left(N_{i}\right)=I_{init}^{T_{m}}-I_{Cur}^{T_{m}+10}$$

(9) $$\Delta V_{T_{M}}\left(N_{i}\right)=V_{init}^{T_{m}}-V_{Cur}^{T_{m}+10}$$

where $T_{m}=0,10,20,30\ldots 1000$, showing that each round consists of 10 s. Equations (7) and (8) show the change in current $\Delta I_{T_{m}}\left(N_{i}\right)$ and change in voltage $\Delta V_{T_{M}}\left(N_{i}\right)$ due to mobility after $T_{m}$ seconds. $I_{init}^{T_{m}}$ shows the initial value of current at $T_{m}$ seconds and $I_{Cur}^{T_{m}+10}$ shows the value of current after $\left(T_{m}+10\right)$ seconds. Similarly, $V_{init}^{T_{m}}$ shows the value of voltage at $T_{m}$ seconds and $V_{Cur}^{T_{m}+10}$ shows the value of voltage after $\left(T_{m}+10\right)$ seconds. The energy consumption $E_{mov}\left(N_{i}\right)$ due to mobility can be rewritten as [48].

(10) $$E_{mov}\left(N_{i}\right)=q_{Mob}d_{Mob}\left(N_{i}\right)$$

where $d_{Mob}\left(N_{i}\right)$ shows the mobility of node $N_{i}$ that is calculated using Euclidean distance after $T_{m}$ seconds. $q_{Mob}$ shows the coefficient of the energy consumption rate. Hence, Equation (6) is derived by merging Equations (7) and (10).

(11) $$E_{Wt\_rd}\left(N_{i}\right)=E_{Mob}\left(N_{i}\right)+E_{Tran}\left(N_{i}\right)+E_{Recv}\left(N_{i}\right),$$

(12) $$E_{Rem}\left(N_{i}\right)=E_{Ini}\left(N_{i}\right)-E_{Wt\_rd(i)}\left(N_{i}\right),$$

where $E_{Wt\_rd}$ is the energy weight, which is assumed as the energy cost of every node after every single round. Hence, $E_{Rem}\left(N_{i}\right)$ is calculated based on Equation (12). $E_{Ini}\left(N_{i}\right)$ is the initial energy of Node$\left(N_{i}\right)$ and $E_{Wt\_rd(i)}$ is the energy weight of node $N_{i}$.

### 3.3. Connectivity

Two mobile sensors nodes $N_{i}$ and $N_{j}$ are connected if they are at $R_{max}$ radio range. Here, a binary disc model is considered [44]. It is the simplest sensing model that is capable of sensing from any point that is located within its sensing radio range and not from any point beyond it. The sensing ability of the mobile sensor node $N_{i}$ is with respect to neighboring node $N_{j}$ within transmission radio range $R_{max}$. The distance $D(N_{i},N_{j})$ between two sensor nodes $N_{i}$ and $N_{j}$ is given by Euclidean distance $D\left(N_{i},N_{j}\right)=\sqrt{{\left(N_{ix}-N_{jx}\right)}^{2}+{\left(N_{iy}-N_{jy}\right)}^{2})}$. Hence, using above equation, the sensing ability is given by.

(13) $$S\left(N_{i},N_{j}\right)=\frac{\Gamma }{D{\left(N_{i},N_{j}\right)}^{Z}},$$

where Γ and *Z* are mobile sensor technology-dependent parameters. The parameter Γ is energy emitted by a target mobile sensor, and *Z* is an energy releasing factor, which ranges from 2 to 5 [49]. The sensor node will transmit “HELLO” packet [50], in a circular area of *R* radius. Using a binary disc sensing model, the node which reply to a “HELLO” packet is accumulated to $NeighN_{i}$ neighboring nodes. Here, we assume a mobile sensor node $N_{j}$ is in $R_{max}$ radio range to sensor node $N_{i}$. If $S(N_{i},N_{j})=1$, then accumulate $NeighN_{i}$ to $NeighN_{i}+1$; otherwise, $NeighN_{i}$ value remains same. This process will continue until $N_{i}$ calculates all its neighbors. Algorithm 1 further explains this process.

**Algorithm 1.** Connectivity algorithm.

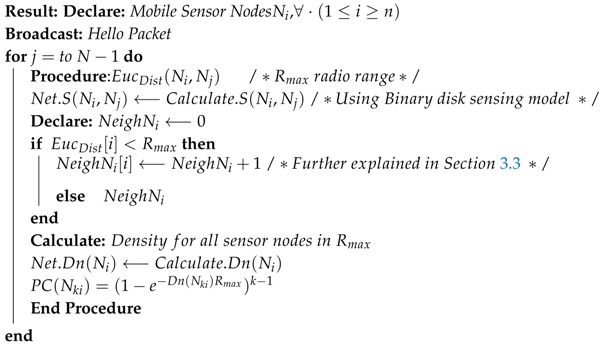



The density of mobile sensor node $N_{i}$ with respect to its neighboring nodes in $R_{max}$ radio range is given by Equation (14) [51], where $Dn(N_{i})$ is density of sensor node $N_{i}$ and ${N_{\pi R}}^{2}$ is the number of nodes located within circular area radio range, which represents the spatial density.

(14) $$Dn\left(N_{i}\right)=\frac{\left(NeighN_{i}\right)}{N_{{\pi R}^{2}}}$$

A node is set to be *k* connected if the removal of k−1 nodes does not disturb its connectivity. Using Equation (15), the probabilistic connectivity of mobile sensor nodes is given by [52].

(15) $$PC\left(N_{ki}\right)={\left(1-e^{-Dn\left(N_{ki}\right)R_{max}}\right)}^{k-1}$$

**Figure 2:**
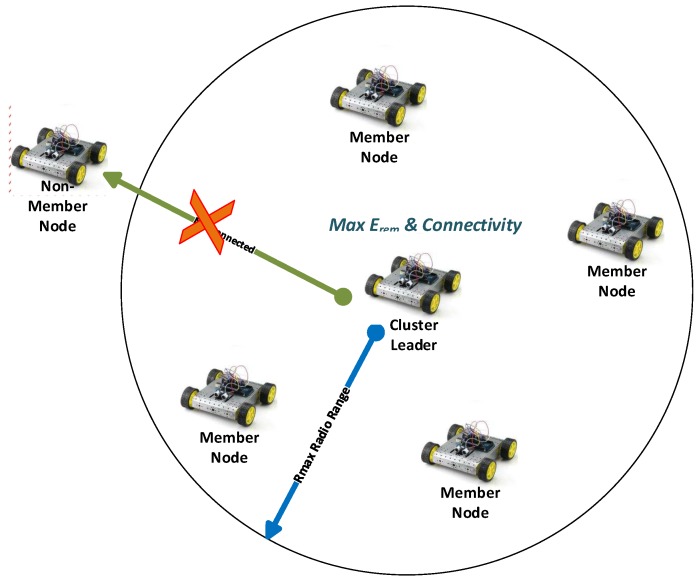
Connectivity model diagram for LEACH-MEEC protocol.

The probabilistic connectivity $PC(N_{ki})$ includes a set of all connected nodes that have at least ki connected sensor nodes in $R_{max}$ radio range. Connectivity is further explained in Figure 2, where circular region is assumed on $R_{max}$ radial range of $N_{i}$ mobile sensor node. Mobile nodes located within $R_{max}$ radial range are considered as neighboring nodes.

Algorithm 1 further presents the steps followed by a mobile sensor nodes to determine connectivity for the selection of CL.

### 3.4. Cluster-Leader Election

In our proposed algorithm, the CL selection process starts with setup phase, where in previous studies CL selection for the first round is carried out as described in [13,16,34,35,39]. During the first round, a mobile sensor node selects a random value between 0 and 1. The selected value should be less than the defined threshold value $O_{thr}\left(x\right)$ calculated by Equation (16).

(16) $$O_{thr}\left(x\right)=\left\{\begin{array}{cc} \frac{p}{1-p*(umod\left(\frac{1}{p}\right))}, & \mathrm{if}\;n\in G\\ 0, & \mathrm{otherwise}, \end{array}\right.$$

where *p* replicates the required percentage of CLs, *n* reflects the number of nodes, *u* is the present round and *G* is the set comprising certain nodes that have not been nominated as CL from last 1/p rounds. After the election of CL, it broadcasts announcement messages to all sensor nodes in neighborhood to become member of its cluster.

From second phase onwards, the CL selection is carried out based on two metrics, remaining energy and connectivity. If a mobile sensor node $N_{i}$ has maximum remaining energy and maximum connectivity within neighboring nodes, it will be elevated for CL selection process. In this paper, we consider connectivity of neighboring nodes within $R_{max}$ radio range for mobile LEACH routing protocol, which, to the best of our knowledge, is a novel approach for mobile LEACH, where CL is selected based on $CV_{i}$ value. All nodes within $R_{max}$ radio range are compared by their $CV_{i}$ values. The one node with maximum value of $CV_{i}$ is selected as CL. $CV_{i}$ is calculated by Equation (17).

(17) $$CV_{i}=E_{Rem}\left(N_{i}\right)+PC\left(N_{i}\right)$$

Hence, the proposed CL selection process is energy efficient, since nodes located outside radio range cannot become part of cluster. The new CL then creates TDMA schedules to accommodate all new sensor nodes and broadcasts it to all cluster nodes in the neighborhood. In steady-state phase, mobile member sensor nodes in a cluster transmit data to CL by turning on their radios in respective time slots. CL transmits the collected data from cluster nodes to the BS. This procedure is repeated until all nodes are dead. Steady-state phase is beyond the scope of this paper.

**Figure 3:**
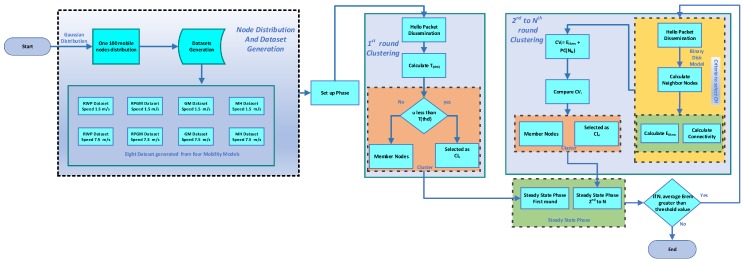
Flow diagram of LEACH-MEEC clustering.

Figure 3 explains a flow diagram of the proposed work. It includes mobile sensor nodes distribution, dataset generation, setup phase (CL selection phase) and steady state phase. This process will repeat until all nodes are dead.

## 4. Simulation Results and Discussion

In this section, simulation environment and datasets, experiments nature, performance parametric and results are discussed in detail. The discussion is based on connectivity feature and its impact on our work. Here, prospect and usefulness is inspected in various simulation environments.

### 4.1. Environment and Datasets

The simulation was carried out assuming a square field that is an obstacle free target area. All simulation experiments were performed in MATLAB (R 2013a) and IBM SPSS (Version-23) was used for statistical analysis. Table 1 shows parametric characteristics that were used for simulation and experimentation. Eight datasets were generated by using four mobility models (RPGM, RWP, GM, and MH). The datasets include one hundred nodes. For individual mobility model, two ranges of speeds were considered: 0.5–1.5 m/s and 5–7.5 m/s. The simulation time duration was 1000 s. Please note that the source code of the simulator is not public yet, but we will share it at request for research purposes under a non-disclosure agreement.

### 4.2. Experiments

This section explains the three types of experiments that were performed to analyze the performance of proposed LEACH-MEEC against three algorithms in the literature, namely, LEACH-MAE, Mobile-LEACH and LEACHDistance-M.

1. Time: The impact of different time duration ranging from 0 to 1000 s over the performance of LEACH-MEEC was studied.
2. Number of Nodes: The impact of different numbers of nodes from 0 to 100 measuring the significance of packet delivery ratio over LEACH-MEEC was studied.
3. Sensitivity Analysis: Different statistical estimation techniques were applied on results to measure the significance of connectivity feature over the performance of our algorithm to strengthen our claim.

### 4.3. Performance Metric

The following three performance metrics were considered for results comparison.

1. Number of Alive Nodes (NAN): The number of remaining alive mobile sensor nodes after *t* seconds of simulation time was measured.
2. Remaining Energy (RE): The average remaining energy (RE) of mobile sensor node at the end of each round was measured.
3. Packet Delivery Ratio (PDR): Packet delivery ratio (PDR) is defined as the ratio between successful delivery of transmitted packets by a source (mobile sensor node) to a destination. The source mobile sensor node receives acknowledgment reply after successful delivery of packets at destination. The performance of protocol is considered better when PDR is high.Thus, we calculated the PDR with Equation (18).
  (18) $$PDR=\frac{PRD}{PTS}$$
  where PDR is packet delivery ratio, PRD is amount of packets received at destination, and PTS is amount of packets transmitted by source.

### 4.4. Results Discussion

The results of LEACH-MEEC were compared with LEACH-MAE, Mobile-LEACH and LEACHDistance-M using the parameters mentioned above.

#### 4.4.1. Number of Alive Nodes (NAN)

**Figure 4:**
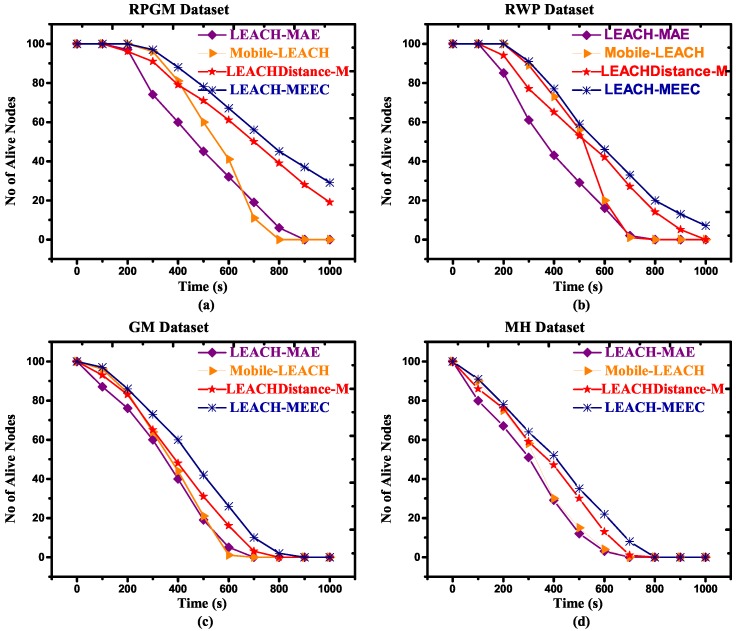
Number of alive nodes versus time for 100 nodes, maximum speed 1.5 m/s, (**a**) RPGM Dataset; (**b**) RWP Dataset; (**c**) GM Dataset; (**d**) MH Dataset.

**Figure 5:**
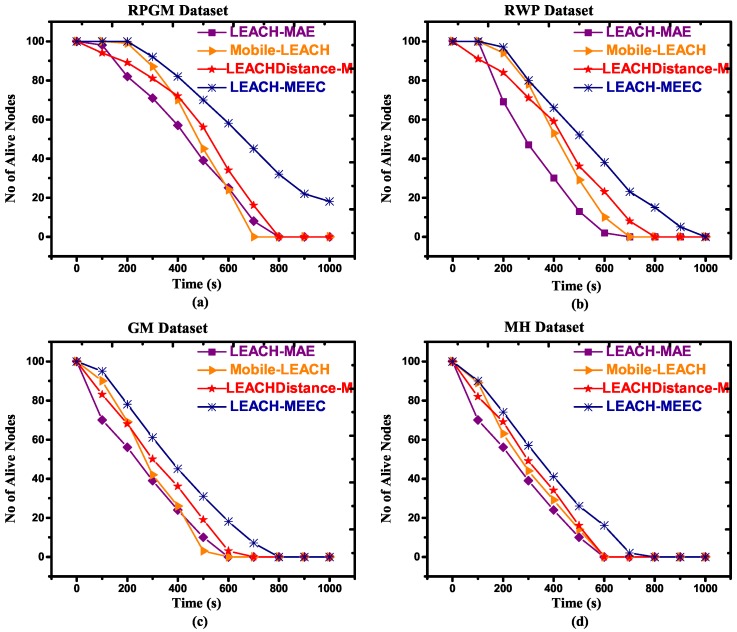
Number of alive nodes versus time for 100 nodes, maximum speed 7.5 m/s, (**a**) RPGM Dataset; (**b**) RWP Dataset; (**c**) GM Dataset; (**d**) MH Dataset.

One of the main benefits of hierarchical clustering is that it improves network lifetime by efficient utilization of energy. The number of alive nodes (NAN) is an important performance parameter for calculating network life time [36]. Figure 4a–d and Figure 5a–d refer to RPGM, RWP, GM and MH datasets with two different speeds, respectively. They show the results comparison of our proposed algorithm with existing routing protocols LEACH-MAE, Mobile-LEACH and LEACHDistance-M based on number of alive nodes. In Figure 4, LEACH-MEEC using RPGM mobility model outperformed LEACH-MAE, Mobile-LEACH and LEACHDistance-M using four mobility models. Our proposed algorithm’s first node die (FND) and last node die (LND) performance is better than all other algorithms. This is primarily because of communication cost of LEACH-MAE, Mobile-LEACH and LEACHDistance-M is much higher as compared to our proposed algorithm. Since LEACH-MAE, Mobile-LEACH selects CL only based on remaining energy, whereas LEACHDistance-M considers distance from base station (base station is located outside area of sensor distribution) and energy to select the CL [40]. In LEACHDistance-M, sensor nodes have high overhead, which reduces network lifetime. In Figure 5, increasing the speed of mobile sensor nodes, the proposed algorithm network lifetime is better than LEACH-MAE, Mobile-LEACH and LEACHDistance-M. The proposed algorithm introduces connectivity feature among the sensor nodes, therefore CL requires less energy consumption to communicate with member nodes, which results in maximum number of remaining alive nodes. RPGM outperforms other mobility models, which can be seen graphically in Figure 5.

#### 4.4.2. Remaining Energy (RE)

**Figure 6:**
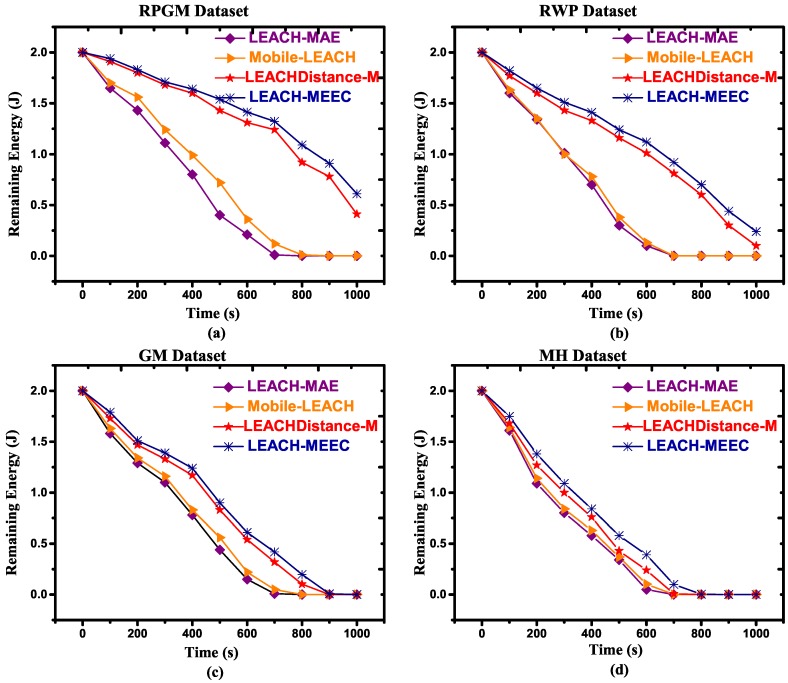
Remaining energy versus time for 100 nodes, maximum speed 1.5 m/s, (**a**) RPGM Dataset; (**b**) RWP Dataset; (**c**) GM Dataset; (**d**) MH Dataset.

**Figure 7:**
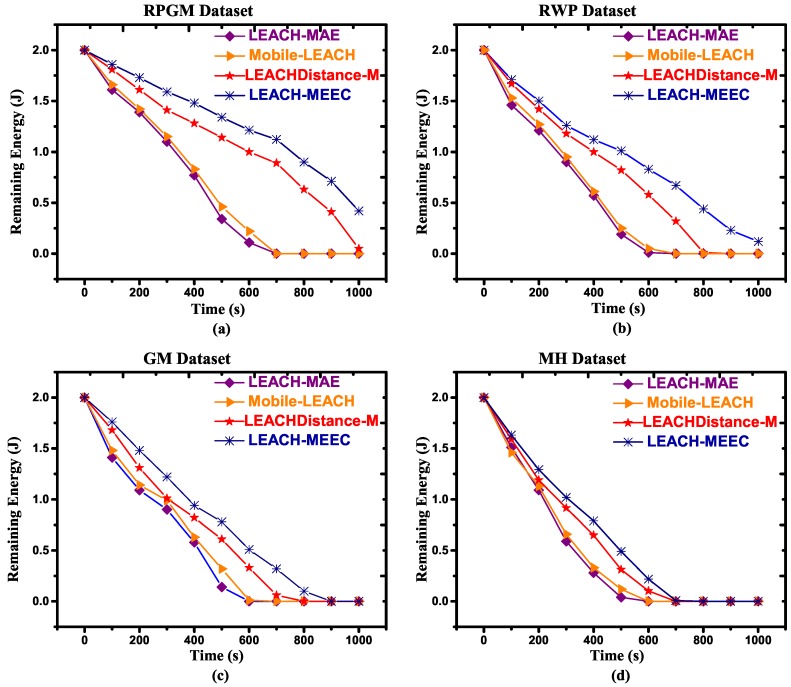
Remaining energy versus time for 100 nodes, maximum speed 7.5 m/s, (**a**) RPGM Dataset; (**b**) RWP Dataset; (**c**) GM Dataset; (**d**) MH Dataset.

In MWSN, energy dissipation rate is higher compared to the static sensor network. In this study, the CL selection was carried out based on connectivity and RE. This criterion prohibits nodes to become CL having maximum remaining energy, but they are far from their member nodes and are not connected, resulting in a long network lifetime. Therefore, RE is an important performance parametric to evaluate the energy efficiency of routing protocols. Our results show that the proposed LEACH-MEEC outperforms LEACH-MAE, Mobile-LEACH and LEACHDistance-M regarding RE. Figure 6a–d and Figure 7a–d refer to RPGM, RWP, GM and MH datasets with two different speeds, respectively. The graphs further explain that RPGM mobility model has maximum RE, since the mobile nodes that are a part of that RPGM have uniform and reflective velocities to the velocity of the group leader similar such as CL. Inter node distance among nodes remain uniform, hence nodes are well connected. It is also pertinent to mention that increases in speed have reduced the energy efficiency, which can be seen in Figure 7a–d.

#### 4.4.3. Packet Delivery Ratio (PDR)

**Figure 8:**
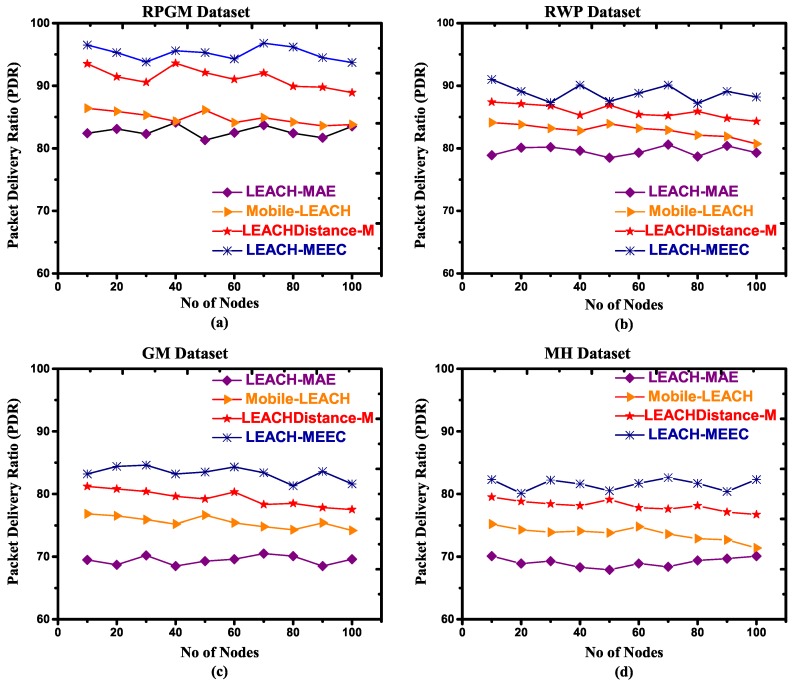
Packet delivery ratio versus number of nodes, maximum speed 1.5 m/s, (**a**) RPGM Dataset; (**b**) RWP Dataset; (**c**) GM Dataset; (**d**) MH Dataset.

**Figure 9:**
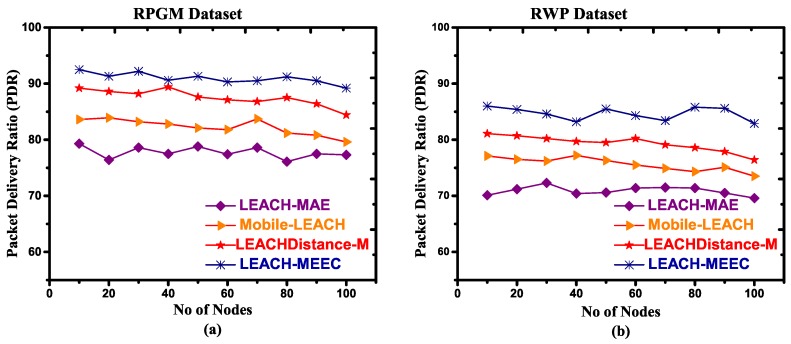

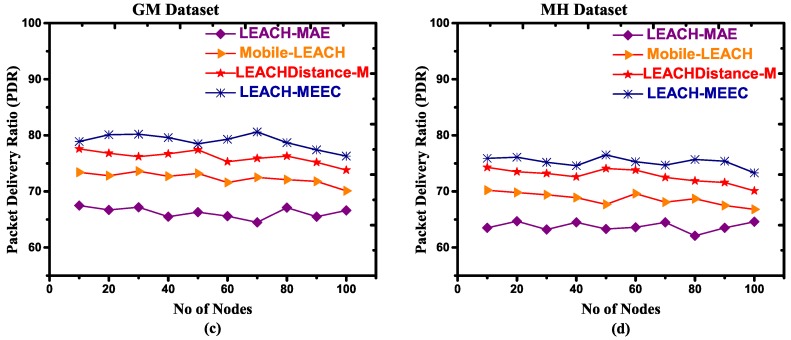
Packet delivery ratio versus number of nodes, maximum speed 7.5 m/s, (**a**) RPGM Dataset; (**b**) RWP Dataset; (**c**) GM Dataset; (**d**) MH Dataset.

MWSN nodes suffer packet loss due to mobility that may result in link collapse. There is an inverse association between speed and packet delivery ratio (PDR) [53]. In RPGM, the mobile sensor nodes are grouped firmly together. Hence, the average inter-node distance among group members is much less than other mobility models. A CL in RPGM can easily receive and deliver messages to member nodes and base station, which reduces the chance of link collapse. Other mobility models have random movement may result in link collapse. Figure 8a–d and Figure 9a–d refer to RPGM, RWP, GM and MH datasets with two different speeds, respectively. Figure 8 shows that LEACH-MEEC PDR is comparatively high as compared to LEACH-MAE, Mobile-LEACH and LEACHDistance-M. The high PDR of LEACH-MEECH is due to an optimal selection of CL. It is also observed that the PDR values fluctuates as transmission distance and speed increase. However, the optimal selection of CL helps the mobile sensor nodes to stay connected due to radio radial connectivity, therefore increasing the sensor network availability and minimizing the packet dropped rate. A comparison for PDR in Figure 9 shows LEACH-MEEC outperforms LEACH-MAE, Mobile-LEACH and LEACHDistance-M using four mobility models datasets.

#### 4.4.4. Sensitivity Analysis

Our simulation results show that the proposed algorithm outperforms existing LEACH-MAE, Mobile-LEACH and LEACHDistanc-M using three performance parameters for four mobility models. To further strengthen our claim, statistical analysis was performed.

(*a*) To find the statistically significant mobility model with respect to the performance parameter of our proposed work. 

**Difference of mean:**Table 2 reports the difference of mean calculated from performance parameter results of our proposed work with respect to four mobility models with average speeds of 1.5 and 7.5 meter per second. Column 1 of Table 2 reports the difference of mean of RPGM with RWP, GM and MH, concerning Average Number of Alive Nodes (ANAN). The result shows that average alive nodes of RPGM are higher than other mobility models and that is statistically significant. Column 2 reports the difference of mean of RPGM with RWP, GM and MH, with respect to average remaining energy. It was found that Average Remaining Energy (ARE) of RPGM based dataset is higher than other mobility models and that is highly significant. Column 3 shows the difference of mean of RPGM with RWP, GM and MH, with respect to average packet delivery ratio (APDR). The result shows that APDR of RPGM mobility model is much higher than other mobility model and that is statistically significant. Similarly, Column 4 compares connectivity metric difference of mean. Here, again, simulation results show that average connectivity (AC) of RPGM mobility model has a high mean difference with comparison to RWP and MH mobility models, whereas it has slightly less mean difference in comparison with GM mobility model, which is still significant. Hence, our proposed algorithm shows better performance with respect to RPGM mobility model dataset than other mobility models that supports our argument.

(*b*) To verify the significant difference of independent variable within the group. 

**Figure 10:**
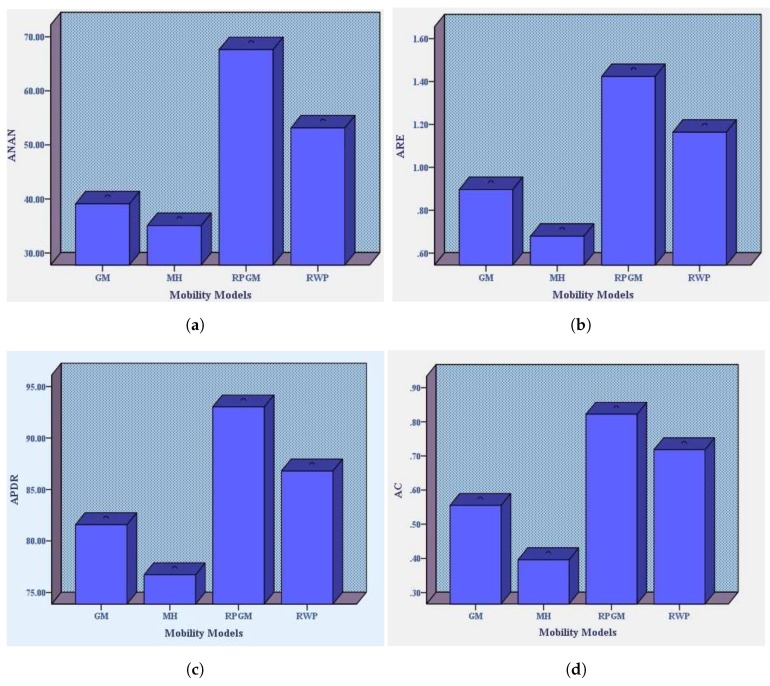
Estimated Means of ANAN, ARE, APDR, AC with respect to Mobility Models. (**a**) ANAN; (**b**) ARE; (**c**) APDR; (**d**) AC.

**One-way Anova and post hoc tests:** We used one-way variance analysis (ANOVA) to check whether RPGM mean is statistically different from other mobility models with respect to four performance parameters. Here, we stated a null hypothesis that “means of all mobility models are equal with respect to four performance parameters”. An alternate hypothesis that at least one of the mobility model’s mean in a group differs. Table 3 reports that all mobility models are different based on four performance parameters. However, it does not tell us individual effect of mobility models and how much one mobility model mean is significantly different from others. Therefore, we conducted two post hoc test (PHT), i.e., Tukey’s test and least square difference (LSD) test. Table 4 shows that the RPGM differs significantly at *p* < 0.05 and it is stronger than other mobility models. Similarly, we further investigated our results by using LSD PHT. It also shows that the effect of RPGM mobility model estimated mean is significantly different. The reason is that RPGM nodes moves in the form of a group. Consequently, it has higher neighborhood connectivity and it balances the remaining energy. Figure 10 shows the performance of mobility models on estimated means of four parameters. RPGM outperforms all other mobility models. It further strengthens our claim that RPGM mobility model performs better with LEACH-MEEC.

(*c*) To perform selection biasness check through Heckman’s two-stage statistical test. 

**Heckman’s two-stage statistical test:** There is a chance that our results are driven by selection biasness. Mostly, selection biasness stems from model or sample biasness. Model biasness is due to leaving out important variable which may have an impact on the dependent variable, whereas sample bias means that our sampling selection procedure may lead to biasness in the model. To address this issue, we use Heckman’s two-stage model [54]. In the first step, we can use probit or logistic regression where RPGM is regressed against ARE and the ANAN with respect to time (seconds). We also add one instrumental variable. That instrumental variable must be correlated to RPGM mobility model but not correlated to connectivity feature. Therefore, we use speed as an instrumental variable. Khan et al. [52] stated there is a direct association between RPGM mobility model and speed. There is no association between speed and connectivity [55,56]. As shown in Table 5, first the probit model was applied to get the value of correction factor inverse mill ratio (λmr), and then the value of (λmr) was added to our main regression model. After adding, it was found that *t*-value of (λmr) is insignificant and the coefficient of RPGM remains positive and significant. Therefore, it shows that connectivity has an impact on the performance of our proposed work.

## 5. Conclusions

The mobility of nodes have many constraints in a WSN, energy efficiency being one of them. This study proposed an improvement in energy efficient LEACH-MAE and Mobile-LEACH. We proposed that the selection of CL is based on two parameters, i.e., remaining energy and probabilistic connectivity among neighboring nodes. We calculated neighborhood connectivity for mobile sensor nodes based on radio radial range. Hence, the improved selection of CL enhance remaining energy of mobile sensor nodes and it improved the life time of network. This study analyzed the proposed algorithm with LEACH-MAE, Mobile-LEACH and LEACHDistance-M using three performance parameters (NAN, RE and PDR). The proposed work outperformed other algorithms while using datasets from four mobility models with respect to different speeds.

Another contribution of this paper is selection of mobility model that is suitable for our proposed work. The results show that the performance of RPGM mobility model is better than that of other mobility models since it has higher connectivity and all the nodes move in the form of group.

To further strengthen our claim, we performed four statistical tests (difference of mean, one-way ANOVA, post hoc (Tukey’s test and LSD) and Heckman’s two-stage test. It was found that the difference of means of RPGM (considering ANAN, ARE, APDR and AC) is statistically significant in comparison with other mobility models. We verified significant difference within and between the groups of mobility models with respect to all performance parameters by applying one-way ANOVA and post hoc (Tukey’s test and LSD). It is proved through both tests that RPGM is more statistically significant within and between the groups as compared to other mobility models. Lastly, we verified through Heckman’s two-stage test that our proposed connectivity parameter is not selection biased. In addition, we found that there is no impact of another instrumental variable. Simulation results and statistical analyses suggest that RPGM mobility model is better for hierarchal clustering in MWSN. In the future, this research work can be extended by increasing the number of mobile sensor nodes and their speeds in a multi-hop environment. We are also working to make the full source code publicly available.

## Figures and Tables

**Table 1:** Simulation parameters.

Key	Value
Field area	100 × 100 m${}^{2}$
Number of MWSN	100
Primary energy of MWSN	2 J
$E_{Tran_{e}lec}$	50 nJ/bit
$E_{Tran_{a}mp_{f}riss}$	10 pJ/bit/m${}^{2}$
$E_{Tran_{a}mp_{2}ray}$	0.0013 pJ/bit/m^4^
One round time	10 s
$d_{m}$	87 m
Maximum velocity	1.5 m/s, 7.5 m/s
Simulation duration	1000 s
Hop	1-hop

**Table 2:** ANAN (Average Number of Alive Nodes), ARE (Average Remaining Energy), APDR (Average Packet Delivery Ratio), AC (Average Connectivity), and D.O.M (Difference of Mean).

	ANAN	ARE	APDR	AC
RPGM	67.62	1.424	93.03	0.82
RWP	53.14	1.1639	86.78	0.73
D.O.M	14.5 **	0.256 **	6.23 ***	0.0872 **
*t*-value	(2.582)	(2.825)	(12.069)	(3.837)
RPGM	67.63	1.424	93.03	0.82
GM	39.098	0.896	81.576	0.555
D.O.M	28.52 ***	0.527 ***	11.43 ***	0.2672 **
*t*-value	(4.86)	(5.062)	(29.863)	(7.47)
RPGM	67.63	1.424	93.03	0.82
MH	35.078	0.6789	76.731	0.396
D.O.M	32.54 ***	0.745 ***	16.28 ***	0.426 ***
*t*-value	(5.55)	(6.907)	(17.001)	(9.77)

Results significant at 1%, 5% are indicated by ***, **, respectively.

**Table 3:** One-way Anova Sum of Squares (S.S).

	ANAN			ARE			APDR			AC		
	S.S	F	Sig	S.S	F	Sig	S.S	F	Sig	S.S	F	Sig
Between Groups	33439.55	11.47	0.000	15.99	5.330	0.000	7479.56	166.89	0.000	5.525	43.32	0.000
Within Groups	194392.16			63.673	0.32		2987.84			8.502		
Total	227831.71			79.66			10467.4			14.027		

**Table 4:** Post hoc tests, one-way Anova, LSD (Least Significant Difference), PHT (post hoc Tests), CMM (Compared Mobility Model), MM (Mobility Model), and M.D (Mean Difference).

			ANAN		ARE		APDR		AC	
PHT	CMM	MM	M.D	Sig.	M.D	Sig.	M.D	Sig.	M.D	Sig.
Tukey HSD	RPGM	RWP	14.49	0.091	0.26	0.096	6.23	0.000	0.104	0.067
		GM	28.529	0.000	0.527	0.000	11.44	0.000	0.267	0.000
		MH	32.549	0.000	0.745	0.000	16.285	0.000	0.426	0.000
LSD	RPGM	RWP	14.49	0.02	0.26	0.021	6.23	0.000	0.104	0.014
		GM	28.529	0.000	0.527	0.000	11.44	0.000	0.267	0.000
		MH	32.549	0.000	0.7445	0.000	16.285	0.000	0.426	0.000

**Table 5:** Heckman’s two-stage statistical test.

Model	RWP	GM	MH
RPGM-RWP	0.065 **		
	(2.248)		
RPGM-GM		0.093 **	
		(1.805)	
RPGM-MH			0.148 **
			(3.632)
ANAN	0.326 **	0.253 **	0.192 **
	(2.701)	(2.964)	(1.982)
ARE	0.628 **	1.838 **	0.682 *
	(5.136)	(13.221)	(2.026)
APDR	0.045 *	0.070	0.150 **
	(1.804)	(1.178)	(2.333)
λmr	0.011	−0.008	0.003
	(0.791)	(−0.438)	(0.021)
Adjusted R2	0.712	0.725	0.742
F-Statistics	62.456 **	66.56 **	72.74 **

Results significant at 5%, 10% are indicated by **, *, respectively.
